# Single-cell transcriptomics reveals biomarker heterogeneity linked to CDK4/6 Inhibitor resistance in breast cancer cell lines

**DOI:** 10.1038/s41523-025-00803-1

**Published:** 2025-07-31

**Authors:** Ilenia Migliaccio, Martina Bonechi, Dario Romagnoli, Giulia Boccalini, Francesca Galardi, Cristina Guarducci, Agostina Nardone, Rachel Schiff, Laura Biganzoli, Luca Malorni, Matteo Benelli

**Affiliations:** 1 Translational Research Unit, Department of Oncology, Hospital of Prato, Azienda USL Toscana Centro, Prato, Italy; 2 Department of Oncology, Hospital of Prato, Azienda USL Toscana Centro, Prato, Italy; 3 Department of Experimental and Clinical Biomedical Sciences “Mario Serio”, University of Florence, Florence, Italy; 4 Lester and Sue Smith Breast Center, Dan L. Duncan Comprehensive Cancer Center, Department of Molecular and Cellular Biology, Department of Medicine, Baylor College of Medicine, Houston, TX USA

**Keywords:** Biomarkers, Transcriptomics, Cancer, Breast cancer, Tumour biomarkers, Tumour heterogeneity

## Abstract

Cyclin dependent kinases 4 and 6 inhibitors have brought great improvements in the treatment of luminal breast cancer, but resistance is a major clinical hurdle. Multiple biomarkers of resistance have been proposed, but none is currently utilized in clinical practice. By performing single-cell RNA sequencing of seven palbociclib-naïve luminal breast cancer cell lines and palbociclib-resistant derivatives, we show that established biomarkers and pathways related to CDK4/6i resistance present marked intra- and inter- cell-line heterogeneity. Transcriptional features of resistance could be already observed in naïve cells correlating with levels of sensitivity (IC50) to palbociclib. Resistant derivatives showed transcriptional clusters that significantly varied for proliferative, estrogen response signatures or MYC targets. This marked heterogeneity was validated in the FELINE trial where, compared to the sensitive ones, ribociclib-resistant tumors developed higher clonal diversity at genetic level and showed greater trascriptional variability for genes associated with resistance. A potential signature of resistance inferred from the cell-line models, positively enriched for MYC targets and negatively enriched for estrogen response markers, was probed on the FELINE trial, separating sensitive from resistant tumors and revealing higher heterogeneity in resistant versus sensitive cells. These data suggest that heterogeneity for CDK4/6 inhibitors resistant markers might facilitate the development of resistance and challenge the validation of clinical biomarkers.

## Introduction

Breast cancer (BC) is a heterogenous disease. Heterogeneity can be observed across different tumors (inter-tumor heterogeneity) or within the same tumor (intra-tumor heterogeneity), in different areas (spatial heterogeneity) or at different times (temporal heterogeneity). It can be detected at genomic, epigenetic, transcriptomic and/or proteomic levels, affecting several aspects of cell functions including proliferation, metabolism, motility, invasiveness and cell-cell interaction. Single-cell sequencing technologies have significantly contributed in dissecting the key role of tumor heterogeneity in BC evolution^1–3^, progression^4,5^, clinical outcomes^6–8^ and drug resistance^9–14^.

The Cyclin Dependent Kinases 4 and 6 inhibitors (CDK4/6i) palbociclib, ribociclib and abemaciclib are the mainstay of treatment for patients with estrogen receptor positive and HER2 negative (ER + /HER2-) metastatic and early high-risk BC^15^. Ongoing clinical trials are also evaluating CDK4/6i in patients with ER+ and HER2 positive (ER + /HER2 + ) BC^16^. However, resistance, either intrinsic or acquired, is a major clinical challenge. Preclinical and translational studies attempted to identify clinically useful markers to direct CDK4/6i treatment^15^ but none are currently evaluated in routine clinical practice.

One of the difficulties in assessing CDK4/6i resistance markers might be related to their high degree of heterogeneity across different samples, clearly inferable by data obtained in vitro^17,18^ in vivo^19,20^, and from patients’ samples^21,22^, with disparate genomic and transcriptomic alterations contributing to CDK4/6i resistance in different settings. As most of the data on resistance to CDK4/6i derive from analyses of bulk population, to date little is known about the role of intra-tumor heterogeneity. Recent studies conducted in models of HER2+ breast tumors resistant to palbociclib plus anti-HER2 therapy and in tumor samples from patients with ER + /HER2- BC treated with ribociclib and endocrine therapy have suggested that intra-tumor heterogeneity might contribute to resistance^14,23^. Therefore, here we analyzed BC models with diverse genomic backgrounds and acquired palbociclib resistance by single-cell RNA sequencing (scRNA-seq) in order to dissect the impact of transcriptional heterogeneity to CDK4/6i resistance in luminal BC.

## Results

### Single-cell transcriptomics of palbociclib-sensitive and resistant cell-lines

We have previously established palbociclib-resistant derivatives (PDR) from seven BC parental models (PDS), by exposing PDS cells to increasing dosing of palbociclib, as explained in the methods section and in our previous publication^17^. PDS models have been chosen to represent a broad range of luminal BC cells, including the ER + /HER2- MCF7, T47D, and ZR751, two endocrine resistant derivatives of MCF7, the EDR and TamR, and two ER + /HER2+ models, the BT474 and MDAMB361. Previous molecular characterization of these models demonstrated a high degree of heterogeneity across models, with very few common markers observed at the time of resistance^17^. To investigate if heterogeneity of resistant markers could be observed also within each PDR model and to assess if we could identify features of resistance already present in parental cells, we performed single-cell RNA sequencing of both PDS and PDR models.

**Fig. 1:**
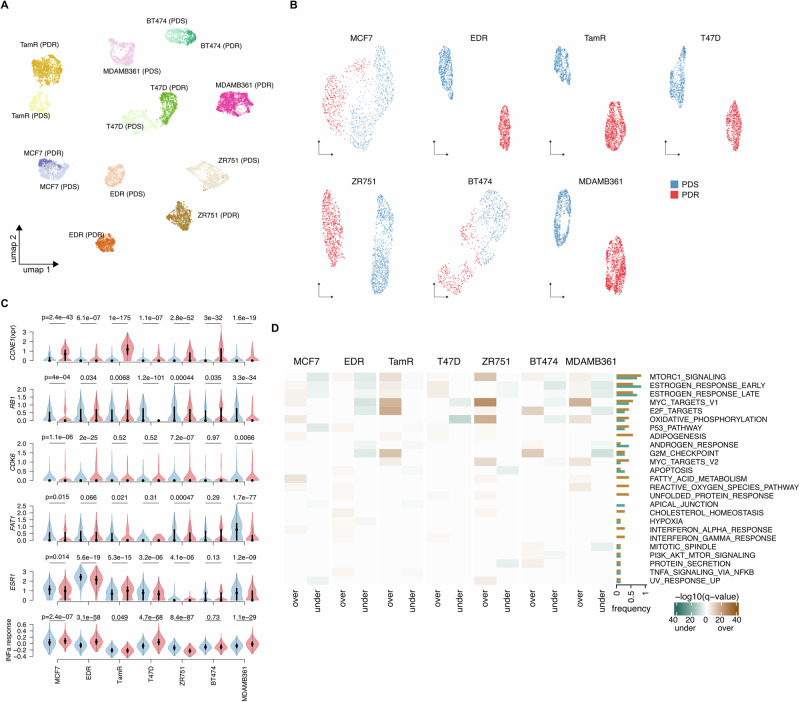
Single-cell transcriptomic characterization of palbociclib resistant breast cancer cell lines. **A** UMAP visualization obtained using the top 5000 most variable genes on all cell lines and **B** the top cell-type differentially expressed genes between palbociclib resistant derivatives (PDR in red) and parental (PDS in blue) models on each cell line. **C** Violin plots of *CCNE1*, *RB1*, *CDK6*, *FAT1, ESR1* and the Hallmark Interferone alpha response signature in sensitive (blue) and resistant (red) cells of the different cell lines. *P*-values are estimated by Wilcoxon test. **D** Heatmap illustrating the significantly enriched Hallmark signatures in the over- or under- expressed genes of PDR versus PDS cells. Over and under indicate higher and lower expression in PDR compared to PDS, respectively. Q-values are estimated using clusterprofiler.

A total of 10,557 cells with at least 2000 genes expressed per cell, corresponding to 5116 parental (PDS) and 5441 resistant (PDR) cells, were selected for downstream analysis. A summary of the metrics related to sequencing, alignment, UMI counts, and gene expression is provided in Supplementary Data 1 while the distributions of the number of cells and genes per cell across the different cell lines are reported in Supplementary Fig. 1. The median genes read was over 3000 for all models and the median number of UMIs per cell ranged from ~3000–4500 across samples, indicating the high quality of the dataset. Considering the 5000 most variable genes, dimensionality reduction analysis based on the uniform manifold approximation and projection (UMAP) algorithm clearly showed segregation of cells based on their cell type, in line with previously generated bulk gene expression data from these cell lines^17^ (Fig. 1A) and consistent with prior scRNA-seq studies in BC^7,11^. However, a secondary segregation between PDR and PDS cells could also be observed, particularly for EDR, ZR751 and MDAMB361 models (Fig. 1A). UMAP performed on each cell line using the top cell-type differentially expressed genes between PDR and PDS models showed a clearer separation between parental and resistant cells for the EDR, TamR, T47D, ZR751, and MDAMB361 models compared to MCF7 and BT474, in which PDR and PDS cells occasionally appeared intermixed with each other (Fig. 1B). These data overall suggest that PDR models have transcriptional features clearly distinguishable from PDS naïve cells using both an unsupervised (most variable genes) and a supervised (top differentially genes) approach.

Several mechanisms and biomarkers of resistance have been identified by us and by others^15,17,19,21,22,24–28^. We analyzed the expression of some previously reported resistance markers, including *CCNE1*, *RB1*, *CDK6, FAT1, FGFR1* and the interferon signaling in our PDR and PDS models (Fig. 1C and Supplementary Fig. 2). As expected, in line with our previous report, all PDR models had a significantly increased expression of *CCNE1* and decreased expression of *RB1*, but the extent of the transcriptional modulations was different across models. *CCNE1* overxpression was higher for the *CCNE1-*amplified TamR PDR and BT474 PDR models^17^ and the *RB1-*deleted T47D PDR and MDAMB361 PDR cell lines^17^ showed lower levels of *RB1* expression. Other markers displayed even greater heterogeneity across models. For example, *CDK6* expression was low across the PDS models, but when levels were compared between PDR and PDS cells, we found that *CDK6* was significantly upregulated in MCF7, EDR and ZR751 and MDAMB361 (Fig. 1C). *FAT1* expression was downregulated in MCF7, TamR, ZR751, and MDAMB361 PDR, but not in the other PDR models (Fig. 1C). *FGFR1* was significantly upregulated in T47D but downregulated in MCF7, TamR, ZR751and MDAMB361 PDR cells (Supplementary Fig. 2). Four PDR models, the MCF7, EDR, T47D and MDAMB361 were characterized by an increased expression of the “Hallmark interferon alpha response” and a previously established signature of interferon pathway activation, the “IFN-Related Palbociclib-Resistance Signature” (IRPS)^27^, compared to PDS. On the other hand, ZR751 PDR had significantly lower levels of these signatures compared to PDS (Fig. 1C and Supplementary Fig. 2). Analyses of cell type markers, such as *ESR1*, *PGR* and *ERBB2* in PDS cells were coherent with what expected for each specific cell line (Fig. 1C and Supplementary Fig. 2). *ERBB2* expression was generally lower in the majority of PDR models compared to PDS, as was *ESR1*, in line with previous reports^21,28^, but a significant increased expression of *ESR1* was observed in the TamR PDR model. A heterogeneous modulation of *PGR* was observed in PDR cells (Supplementary Fig. 2).

Enrichments of the Hallmark signatures in differentially expressed genes between PDR and PDS cells (over: enrichment resulting from genes with higher expression in PDR versus PDS, under: enrichment resulting from genes with lower expression in PDR versus PDS), confirmed the high degree of heterogeneity across PDR models (Fig. 1D and Supplementary Data 2), with each model showing distinctive enrichment patterns. We found that the most commonly significantly (*q* < 0.05) deregulated terms were “Hallmark MTORC1 signaling” and “Hallmark estrogen response early”, resulting enriched from both over- and/or under-expressed genes depending on cell type. For examples, MCF7 PDR cells showed enrichment of the “Hallmark MTORC1 signaling” in under-expressed genes and enrichement of the “Hallmark estrogen response early” in both over- and under-expressed genes. On the other hand, T47D showed enrichment of both terms in over-expressed genes. Overall these data highlight how markers and pathways of resistance might differ across cell type, possibly due to different underlying mechanisms.

### Heterogeneity of palbociclib resistance markers within models

**Fig. 2:**
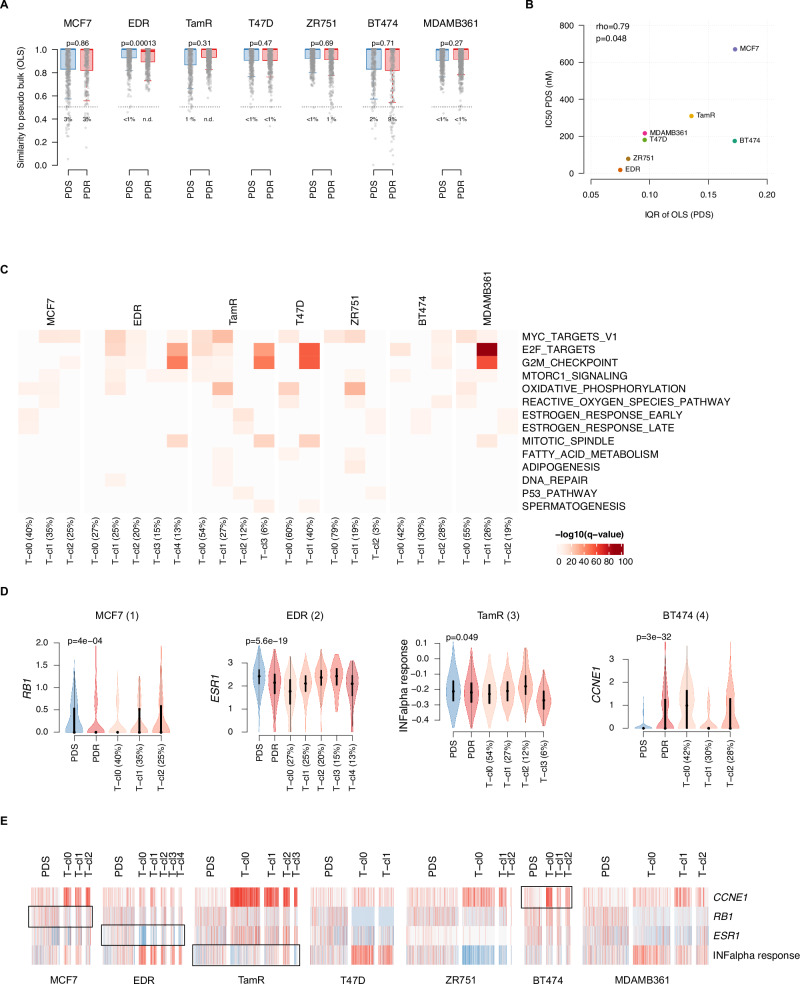
Intra-tumoral heterogeneity in resistance to CDK4/6i. **A** Box plots showing the distribution of the similarity to the cell-type specific differentially expressed genes derived from the pseudo-bulk calculated by Ordinary Least Square (OLS) in palbociclib resistant derivatives (PDR in red) and parental (PDS in blue) cells. *P* values are estimated by Wilcoxon test. **B** Scatter plot showing the inter quartile range (IQR) of the ordinary least squares (OLS) and the IC50 values for palbociclib (nM) in parental (PDS) cells. *P* value is estimated by Spearman’s correlation test. **C** Heatmap showing the significantly enriched Hallmark signatures, identified from the differentially overexpressed genes, among PDR transcriptional clusters for each cell lines. Q-values are stimated from clusterprofiler^55^. **D** Violin plots showing the distribution of resistance-associated markers in selected cases revealing intra-tumor heterogeneous distribution. *P*-values are stimated from Wilcoxon test. **E** Heat map showing the expression levels of the resistance-associated markers *CCNE1*, *RB1*, *ESR1* and “Hallmark interferone alpha response” in PDS cells and PDR cells grouped according to transcriptional clusters.

To assess if the sensitivity/resistance features could help correctly predict PDR and PDS cells and to estimate heterogeneity of these features within the PDS and PDR populations, we applied the ordinary least squares (OLS) approach^29^ at each cell line, using the cell-type specific differentially expressed genes derived from the pseudo-bulk data. In general, in all models, the majority of cells were correctly predicted as PDS or PDR (Fig. 2A). However, different levels of OLS were observed both within PDS and PDR cells. Interestingly, using the OLS cut-off of 0.5, in all PDS models we could identify subpopulations of cells predicted as more similar to the PDR pseudo-bulk (PDR-like), although the proportion of PDR-like cells was different across models and higher for MCF7 (3%), TamR (1%) and BT474 (2%) (Fig. 2A) models in which OLS also highlighted higher heterogeneity. Of note, we also found that parental PDS cells with higher heterogeneity, as measured by the inter quartile range of OLS, had significantly higher IC50 values for palbociclib (rho = 0.79, *p* = 0.048, Fig. 2B). These data suggest that transcriptional features of palbociclib resistance can be observed in PDS cells and that heterogeneity for resistant markers might affect sensitivity to CDK4/6i. To investigate the molecular features underlying the PDR-like phenotype, we analyzed differentially expressed genes between PDR-like and PDS cells in MCF7, TamR and BT474 models. We found that they significantly correlated with differentially expressed genes between pseudo-bulk PDR and PDS cells (Supplementary Fig. 3). However, enrichment analysis of differentially expressed genes in PDR-like cells did not show commonly enriched signatures across the different models, but revealed features consistent to the corresponding PDR models (Supplementary Data 3). For example, over-expressed genes of MCF7 PDR-like cells were enriched for the interferon signatures, in line with MCF7 PDR cells, while, in accordance with what was observed in BT474 PDR cells, the PDR-like BT474 cells showed enrichment of the “Hallmark estrogen response early” in the underexpressed genes. These data suggest that pre-existing resistant features are cell-type specific and heterogenous across models. It was previously demonstrated that pre-adapted BC cells might be found when cells are still sensitive to endocrine therapy and a signature of pre-adaptation has been derived from these cells^10^. We assessed the levels of this signature in PDR-like cells. When considering all models together, we found a significant upregulation of genes upregulated in pre-adapted cells (Supplementary Fig. 4A) in PDR-like compared to PDS-like cells. However, due to the high variability found across models and the very low number of cells in some of the models (Supplementary Fig. 4B), no strong conclusions could be drawn from this observation.

To further explore the heterogeneity for CDK4/6i resistance transcriptional features within the PDR population, we performed clustering analysis by Louvain algorithm on each PDR model using the top variable genes filtered for the cell-type differentially expressed genes in resistant versus sensitive cells. PDR cells were characterized by a variable number of transcriptional clusters (T-cl), ranging from 2 in the T47D to 5 in the EDR (Supplementary Fig. 5). Interestingly, in each PDR model, the T-cl showed different distribution of PDR-like similarity by OLS (Supplementary Fig. 6A), different proportion of cells classified according to PAM50 subtypes (Supplementary Fig. 6B) and of cells in the different cell cycle phases (G1, G2/M and S) (Supplementary Fig. 6C), but a high degree of overlap was observed only for few clusters, suggesting that these features are not major contributors to the T-cl. We also estimated large-scale chromosomal copy number (CN) alterations and clusters (C-cl) according to InferCNV and assessed their overlap with transcriptional clusters, finding a high degree of overlaps only for some of the ZR751 and BT474 PDR clusters (Supplementary Fig. 6D), suggesting that T-cl are overall independent from CN alterations.

To analyze the source of intra-tumor heterogeneity, we performed an enrichment analysis of the Hallmark signatures in the differentially expressed genes among the T-cl of PDR models (Supplementary Data 4). Significantly enriched terms identified from the over-expressed genes in each cluster are shown in Fig. 2C. Notwithstanding the heterogeneity across cell models, we could identify three main patterns of enrichments: clusters mainly enriched for the proliferation signatures “Hallmark E2F targets” and “Hallmark G2M checkpoints” (e.g., T-cl 4 in EDR, T-cl3 in TamR and T-cl1 in T47D and MDAMB361); clusters mainly driven by the ER signaling, being enriched for “Hallmark estrogen response early” and “Hallmark estrogen response late” (e.g., T-cl0 in MCF7, T-cl2 in TamR and ZR751 and MDAMB61 and T-cl1 in BT474); and clusters enriched for metabolic terms like “ Hallmark oxidative phosphorylation” and “Hallmark fatty acid metabolism” which were also enriched for the proliferation signature “Hallmark MYC Targets V1” and for the “Hallmark MTORC1 signaling” (e.g., T-cl1 in MCF7, EDR, TamR and ZR751, T-cl0 in T47D and MDAMB361 (Fig. 2C).

To assess the heterogeneity of well-established markers of resistance within PDR models, we analyzed the expression of *CCNE1*, *RB1*, *ESR1* and the “Hallmark interferon alpha response” of the T-cl. Figure 2D illustrates interesting examples of heterogeneity for each of the markers analyzed while Fig. 2E shows the expression of these markers in PDS cells or PDR cells grouped according to the T-cl for each model. T-cl2 of MCF7 did not show the downregulation of *RB1*, and the downregulation of *ESR1*, observed in the overall population of EDR, was observed mainly in some of the clusters. The TamR PDR model is characterized by an overall reduced expression of the “Hallmark interferon alpha signaling”. However, in this models, the T-cl2 showed an over-expression of this signature compared to the other T-cl. T-cl 1 of the BT474 PDR, a *CCNE1* amplified model, was characterized by low expression levels of *CCNE1*, comparable to those in the BT474 PDS cells.

### Evaluation of heterogeneity for CDK4/6i resistance markers in clinical samples

The FELINE trial assessed the CDK4/6i ribociclib in addition to endocrine therapy in the neo-adjuvant setting. Patients with ER + /HER2- early staged BC were randomized to receive: arm A) endocrine therapy alone (letrozole plus placebo), arm B) intermittent high dose ribociclib (600 mg/d) plus letrozole or arm C) continuous lower dose ribociclib (400 mg/d) plus letrozole. Patients were treated for six cycles, and biopsies were collected at baseline (Day 0), following 14 days of treatment (Day 14), and at the end of treatment (surgery around Day 180). Patients were classified as sensitive or resistant based on response, and a subset of serial biopsies was examined by scRNA-seq^14^.

**Fig. 3:**
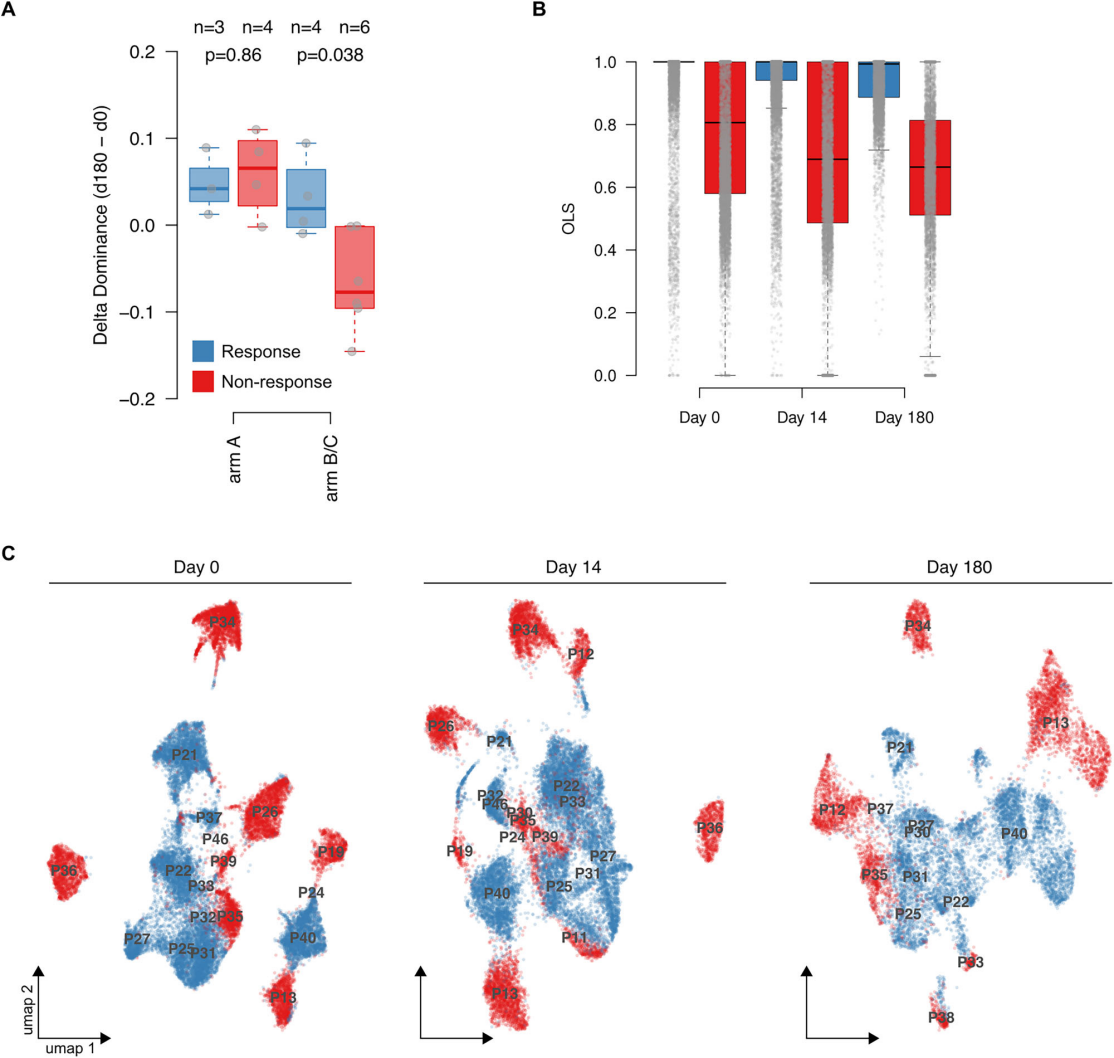
Heterogeneity of CDK4/6i resistance markers in the FELINE dataset. **A** Box plots showing the distribution of the delta dominance at day 180 compared to day 0 divided according to patients’ response to letrozole alone (arm A) or ribociclib and letrozole (arm B/C). The scores were derived from the original manuscript^14^. *P* values were estimated by Wilcoxon test. **B** Box plots showing the distribution of the similarity to pseudo-bulk population calculated by Ordinary Least Square (OLS) in ribociclib resistant (red) versus sensitive (blue) cells using the differentially expressed genes derived from the comparison of resistant versus sensitive tumors at baseline (Day 0). **C** UMAP visualization of the scRNA-seq FELINE dataset obtained using the top variable genes filtered according to the PDR markers derived from our cell line dataset and including patients treated with ribociclib, independently of schedule (Arm B + C) at day 0, day 14 and day 180. Red= resistant patients; blu= sensitive patients.

It was previously demonstrated that cells from FELINE patients treated with the combination of ribociclib and letrozole showed increased intra-tumor heterogeneity during treatment compared to those treated with letrozole alone^14^. The analysis of the heterogeneity from the Whole Exome Sequencing of tumor samples according to the sensitivity/resistance status revealed an increased heterogeneity as measured by subclonal dominance in ribociclib-resistant compared to sensitive patients (Fig. 3A, the other heterogeneity meaures are reported in Supplementary Fig. 7), while no difference was observed in sensitive/resistant patients treated with letrozole, suggesting that heterogeneity might contribute to acquired resistance, specific to CDK4/6i.

We accessed the publicly available scRNA-seq dataset derived from FELINE in order to further depicting the heterogeneity of the single cell population for the CDK4/6i resistant markers and to examine the clinical relevance of markers derived from our cell-line dataset.

To assess heterogeneity for the CDK4/6i resistant markers, we first identified differentially expressed genes between samples from sensitive and resistant patients (pseudo-bulk analysis) at baseline (Day 0) and then applied the OLS approach to estimate heterogeneity of the sensitivity/resistance features within the single cell populations. At all time points, we found significantly less similarity to the pseudo-bulk population among the resistant cells compared to sensitive, suggesting higher heterogeneity for the resistant markers within the resistant population (Fig. 3B).

We next aimed to select, from our cell models, markers that could help further investigating heterogeneity in clinical samples from the FELINE trial. Therefore, we identified genes (*n* = 900) that were specifically up-regulated (*n* = 423, PDR specific) or down-regulated (*n* = 477, PDS specific) in at least two PDR models compared to their PDS counterparts (Supplementary Data 5), from now on called PDR markers when considering PDR and PDS specific together. Enrichment analyses (Supplementary Data 6) of the PDR specific markers showed the “Hallmark MYC Targets V1” as the top enriched signature among the hallmarks datasets, while the “Hallmark Estrogen Response Early” was the top Hallmark signature enriched in the PDS specific markers. Of note, at all time points, PDR markers were significantly enriched in the list of differentially expressed genes between cells derived from patients sensitive and resistant to ribociclib in the FELINE trial (proportion test, Supplementary Fig. 8A). We performed clustering analysis considering the top variable genes of the dataset and then filtering them by the PDR markers. When considering the top variable genes, UMAP showed a prevalent separation of the tumor cell population according to patient identity, but also a secondary distribution according to resistance/sensitivity status (Supplementary Fig. 8B). The selection of top-variable genes by PDR markers showed a more evident segregation according to the sensitive and resistant status at all time points, with patients identity only displaying a secondary contribution (Fig. 3C). To confirm this observation, we calculated, at all time points, the euclidean distance among resistant and sensitive cells of the FELINE trial using the top variable genes filtered by the PDR markers (*n* = 95) or by randomly selected genes of the same size (*n* = 95) as PDR markers (see Methods). In line with the UMAP data, we found a greater distance when using randomly selected genes than PDR marker, suggesting that PDR markers induced a better clusterization of the cells according to the sensitive/resistant status (Supplementary Fig. 8C). In addition, PDR markers showed greater distance in the resistant population compared to sensitive at all time points, suggesting greater heterogeneity of resistant cells for these markers (Supplementary Fig. 8C).

## Discussion

CDK4/6i have brought significant improvements in the treatment of metastatic and early high-risk BC, but it is now clear that some patients do not benefit from these agents, showing de novo resistance, and even the responders eventually recur, experiencing acquired resistance. Numerous preclinical and clinical studies investigated the determinants of both de novo and acquired resistance with the aim of identifying clinically useful biomarkers, but to date, only ER positivity and HER2 negativity are used to direct CDK4/6i therapy^15^. Analyzing different in vitro BC models with acquired resistance to CDK4/6i, we and others demonstrated the difficulties in finding a unifying marker of resistance^17,18^. In our models, the most consistent markers were the overexpression of Cyclin E1 and underexpression of Rb, albeit with different levels across models, but the mechanisms leading to the modulations of these markers are likely heterogeneous, as mutations or CNA of the corresponding *CCNE1* and *RB1* genes cannot be demonstrated in all lines^17^. Therefore, in the present manuscript, we primarily focused on assessing the impact of heterogeneity of CDK4/6i resistance markers in BC models and patient samples, as sparse data exist on this topic.

By analyzing selected markers of CDK4/6i resistance and performing enrichment analyses on differentially expressed genes between PDS and PDR cells, we could confirm the heterogeneity for most of the previously described CDK4/6i resistance pathways across our models.

By assessing features of sensitivity and resistance using OLS, we found that cells with transcriptional features of resistance could be already detected at the time of sensitivity, albeit only a very small proportion of cells classified as PDR-like could be observed in the more homogenous models. The presence of PDR-like cells in all models raises the question of whether resistance to palbociclib is more likely to occur through selection of pre-existing cells/features and whether targeting resistance mechanisms upfront using combinatorial strategies might be an effective strategy in preventing the development of acquired resistance. However, more data are needed.

In this study, we also demonstrated that cells resistant to palbociclib are characterized by high transcriptional heterogeneity for resistant biomarkers and pathways. We found that, despite the overlay between some of the T-cl and the cell cycle phases or the CN clusters, the majority of the T-cl were not overlapping. For this study, we decided not to mitigate the effects of cell cycle heterogeneity since cell cycle is the main target of CDK4/6i and we could have masked some of the drug effects.

In most of our PDR models, the determinants of transcriptional heterogeneity seemed to be predominantly related to proliferation, estrogen response and mTOR/metabolic pathways/MYC targets, all pathways that have been previously associated to CDK4/6i resistance^14,17,25,30–33^ suggesting that different mechanisms might coexist in the same tumor. In addition, analyzing resistant markers like *RB1*, *ESR1*, *CCNE1*, and the “Hallmark Interferone alpha response “ signature we could appreciate a heterogeneous distribution across clusters for these markers. This might be of potential clinical relevance, if confirmed in clinical samples. Indeed, the absence of *CCNE1* overexpression/amplification in a subpopulation of cells within a *CCNE1*-amplified bulk tumor could be an escape mechanism for therapies targeting this gene. A reduced expression of the interferon signaling at the time of resistance does not exclude the presence of an interferon-overexpressing subpopulation, as shown in the TamR PDR model. On the other hand, it must be acknowledged that other markers of resistance were quite homogenous in some of the models. For example, *RB1* expression was completely lost in all T47D PDR cells, displaying a genetic loss of *RB1*.

As a proof of principle, we applied a selection of markers derived from our models to the FELINE trial^14^ to demonstrate their potential applicability in a clinical setting. We acknowledge that the ribociclib-resistant cells from the FELINE trial might not be the optimal dataset to validate our palbociclib-based findings, as some differences in mechanism of resistance between ribociclib and palbociclib were described^34,35^. Nevertheless, we were still able to demonstrate that PDR markers were significantly enriched in differentially expressed genes between patients resistant to ribociclib versus sensitive and were able to discriminate resistant patients at all time points suggesting that, despite being derived from palbociclib resistant cellular models, they might still be relevant in ribociclib treated clinical samples. In addition by PDR marker and OLS analysis we highlighted higher heterogeneity for the resistant markers in the ribociclib-resistant cells compared to sensitive and we observed increasing heterogeneity in the resistant population compared to the sensitive in patients treated with ribociclib plus endocrine therapy, but not in those treated with endocrine therapy alone at all timepoints. This result adds to previous data from the original publication of the FELINE trial, which showed higher heterogeneity during combination compared to endocrine treatment, and suggests that heterogeneity might be more relevant during the development of CDK4/6i resistance and might potentially impact on the identification and validation of biomarkers of resistance/sensitivity.

A better understanding of the mechanisms leading to heterogeneity during CDK4/6i treatment is advisable. Heterogeneity might rely on the deregulation of DNA repair mechanisms and the increased DNA damage, previously described in cellular models and clinical samples resistant to CDK4/6i^18,36^. Alternatively, being CDK4/6-Rb axis downstream to different pathways, mechanisms of resistance might differ depending on the cellular and or molecular context. However, more experimental data are needed.

We acknowledge that one of the limitations of our work might be related to the relatively low number of cells (ranging from around 400–1300) isolated and sequenced by Bio-Rad ddSEQ, which might have limited our ability to capture the full spectrum of heterogeneity within each sample. However, even the cell lines in which less cells were analyzed, the MCF7 and BT474, were indeed highly heterogenous. Furthermore, ddSEQ enabled the analysis of a larger number of genes per single cell when compared to other state-of-the-art technologies, thereby enabling the analysis of genes expressed at low to moderate levels. It must be also underlined that the number of cells analyzed does not differ from that of previous remarkable scRNA-seq studies investigating the heterogeneity, evolution and response to treatment in BC^6,9,13,37^.

Another limitation might be related to the use of BC cell lines as models of heterogeneity. Indeed, it has been demonstrated that continuing genomic instability of cells might impact on their levels of heterogeneity^38^. While this holds true, it must be considered that BC cell lines have significantly contributed to improve our knowledge in BC biology and drug resistance. Large cell lines datasets demonstrated that they can recapitulate the heterogeneity observed in patients in terms of genomic alterations, BC subtypes and drug response^39^, including CDK4/6i sensitivity^40^. Furthermore, it was recently shown that the evaluation of intra-tumor heterogeneity of BC cell models might provide useful information in predicting drug responses^41^.

In our study we analyzed different models of ER+ BC, including ER+ endocrine resistant cells and ER + /HER2+ cell models. Our analyses reveal that each model behaves uniquely in terms of transcriptional and genomic variations and might bring important information regarding the development of resistance. Our dataset might be of help in generating or confirming hypotheses on acquired resistance to palbociclib and in the assessment of heterogeneity of resistance markers, which might ultimately assist to better personalize treatment of patients with ER + BC.

## Methods

### Cell lines

Seven luminal BC cell lines were analyzed, including three ER + /HER2- models, MCF7, T47D and ZR751, two endocrine-resistant derivatives of MCF7 resistant to estrogen deprivation, the MCF7/EDR (EDR), or to tamoxifen, the MCF7/TamR (TamR), and two ER + /HER2+ models, BT474 and MDAMB361. For all seven models, derivatives resistant to palbociclib have been established^17^. Full information on the parental (PDS) and resistant (PDR) cells and the methodology used to develop PDR derivatives have been previously published^17^. Briefly, MCF7, T47D, ZR751, BT474 and MDAMB361 parental cells were cultured in Dulbecco’s modified Eagle’s medium (DMEM) with 4.5 g/glucose and L-glutamine supplemented with 10% heat-inactivated fetal bovine serum (FBS) and 10,000 U penicillin and streptomycin 10 mg/mL solution (P/S). Parental EDR and TamR were grown in DMEM without L-glutamine and phenol red supplemented with 10% charcoal-stripped FBS and P/S. TamR medium was also supplemented with 100 nM of (Z) − 4-Hydroxytamoxifen. PDR derivatives were established by culturing parental cells with increasing concentration of palbociclib and were deemed resistant when they restored their growth in the presence of 1 μM of palbociclib; thereafter, PDR medium was supplemented with palbociclib 1 μM. The half-maximal inhibitory concentrations (IC50 values) for palbociclib of PDR and PDS cells have been previously assessed and reported^17^.

### Single-cell isolation and sequencing

Methods for the single cell isolation, library preparation and sequencing have been previously published^42^. Briefly, cells at a confluency of about 60–90% were detached by Trypsin, checked for viability, counted using the Countess^TM^ automated cell counter (Invitrogen, Thermo Fisher) and diluted to obtain the target concentration of 2500 cells/μl. Viability of >95% was required according to the manufacturer’s instructions. Libraries were prepared by a specific Illumina kit, the SureCell WTA 3’, according to the manufacturer’s instructions. Cell Mix and Barcoded Mix were prepared and loaded into the ddSEQ cartridges to generate the single cell droplets by Bio-Rad ddSEQ single cell isolator, which allows the isolation and barcoding of about 300 cells per well^43^. For each cell line, at least two wells for the PDS and two wells for the PDR derivatives were used. The cDNA libraries were assessed on the Agilent 2100 Bioanalyzer using the High sensitivity DNA chips and sequenced on HiSeq 2500 Illumina platform in order to obtain about 100,000 reads per cell.

### scRNA-seq data processing

Reads were processed with ddSeeker^42^ to extract molecular and cellular tags, and further analyzed with Drop-seq tools (v1.13)^44^ to perform the following operations: alignment of tagged reads to the reference genome hg38 using STAR (v2.6)^45^; filtering, sorting and merging of BAM files using GATK (v4.0.7)^46^; creation of the expression matrix reporting the number of reads for each gene, in each cell, for each sample. Results from a preliminary analysis on the reads from the MDAMD361 parental cells by the ddSeeker tool have been previously published^42^ demonstrating the ability to identify valid reads for downstream analyses.

### Analysis of PDS and PDR cell-line scRNA-seq data

Genes were considered expressed if relative expression by Monocle^47^ was 0.1 or more in ten cells or more. Only cells with at least 2000 and at most 7000 expressed genes, and with no more than 10% of counts mapping to mitochondrial genes, were considered for downstream analyses. Seurat version 4.3.0^48^ was used for counts normalization, clustering, differential expression and dimensionality reduction analyses. To focus on messenger RNA, mitochondrial and ribosomal genes were excluded from all analyses. For clustering, dimensionality reduction and differential expression analysis, counts normalization was performed using sctransform^49^, using the percentage of mitochondrial genes as covariate. Dimensionality reduction analysis by UMAP algorithm was applied on the first 20 principal components identified by Principal Component Analysis. To identify the most appropriate number of clusters detected using the Louvain algorithm and therefore estimate the optimal resolution, the multiK^50^ R package version 0.1.0 was applied to each sample (nPC = 30, nreps = 100), varying resolution in steps of 0.1, ranging from 0.1 to 2. Cell-cycle phases were estimated by cyclone^51^ implemented in scran^52^ R package version 1.26.2. PAM50 classes were estimated by genefu R package version 2.30.0^53^, assigning PAM50 classification only for those cells showing corresponding class coefficient > 0.7. Gene-expression signatures were estimated by GSVA^54^ version 1.46.0. Functional enrichment analysis was performed with clusterProfiler version 4.6.0 using HALLMARK version 7.4^55^.

### Estimation of PDS-PDR similarity

To calculate the similarity to PDS and PDR cell lines, we applied an OLS^29^ method to the list of cell line-specific PDR versus PDS genes. Specifically, for each cell line, we considered the list of genes with an absolute log2 fold-change of 0.5 or more in PDR versus PDS, using our previously published gene expression data^17^, with the additional condition of being also amongst the top 2000 most variable genes across PDR and PDS scRNA-seq data.

### Identification of PDS and PDR marker genes

For each cell line, differential gene expression analysis between PDR and PDS was conducted using FindAllMarkers function implemented in Seurat (default parameters except for min.pct = 0.25 and logfc.threshold = 0.25). Genes detected as over-expressed in PDR versus PDS cells in at least two cell lines and never detected as over-expressed in PDS versus PDR cells were defined as PDR-specific. Genes detected as over-expressed in PDS versus PDR cells in at least two cell lines and never detected as over-expressed in PDR versus PDS cells were defined as PDS-specific.

### Analysis of inferred CN alterations

InferCNV version 1.14.2 was used to estimate large-scale CN Alterations (https://github.com/broadinstitute/inferCNV.) using hormone-responsive luminal cells (termed L2) as reference cells, as previously reported^10^, from log-normalized gene expression data estimated by Seurat. Subclusters were estimated by the Leiden algorithm.

### Analysis of scRNA-seq data from the FELINE study

FELINE scRNA-seq counts were downloaded from Gene Expression Omnibus (GEO) (accession number: GSE158724). For downstream analysis, we selected cells in which a minimum of 500 genes were detected, resulting in *n* = 110,558 cells and *n* = 34 patients. Counts normalization was performed using sctransform^49^. For cluster and dimensionality reduction analysis, for Supplementary Fig. 7B we considered the top 2000 most variable genes identified by Seurat, while for Fig. 4C the intersection between those genes and the list of PDS or PDR marker genes, depending on the specific analysis. Differentially expressed genes between sensitive and resistant patients were identified by FindMarkers using default parameters in baseline samples (Day 0). To estimate heterogeneity in sensitive and resistant patients (Supplementary Fig. 7C), we calculated pairwise euclidean distances among 1000 randomly selected cells based on the normalized gene expression level of the 95 PDS and PDR marker genes within the top 2000 variable genes (marker), and 100 random selections of 95 genes from the top variable genes, excluding the 95 PDS and PDR marker genes (topvar). For the top variable genes, distances were computed as the average pairwise distance across the 1000 randomly selected cells.

## Supplementary information

Supplementary Information

Supplementary Data

## Data Availability

The scRNA-seq count data matrix generated in this study are available in GEO, accession code GSE298567.

## Code availability

The code to reproduce the analyses presented in the manuscript is available upon request.
