# Supplementary material for: Single-cell transcriptomics reveals biomarker heterogeneity linked to CDK4/6 Inhibitor resistance in breast cancer cell lines
Source: NPJ Breast Cancer. 2025 Jul 31;11:82. doi: 10.1038/s41523-025-00803-1 (PMC12311131; doi:10.1038/s41523-025-00803-1)
Supplement: Supplementary file 1 — Supplementary Information [file 41523_2025_803_MOESM1_ESM.pdf]

Supplementary Information to:

Single-Cell transcriptomics reveals biomarker heterogeneity linked to CDK4/6 Inhibitor resistance in breast cancer cell lines

List of Supplementary Data

Supplementary Data 1. Summary of Sequencing, Alignment, and Count Statistics of the scRNA-seq Samples

Supplementary Data 2. Functional enrichment analysis by clusterProfiler of cell-line specific differentially expressed genes between PDS and PDR

Supplementary Data 3. Functional enrichment analysis by clusterProfiler of differentially expressed genes between PDS PDR-like cells and the other PDS cells

Supplementary Data 4. Functional enrichment analysis by clusterProfiler of genes identified as differentially expressed in each transcriptonal cluster (T-cl) by Seurat and MultiK across all cell lines

Supplementary Data 5. List of 900 PDR markers detected across cell lines

Supplementary Data 6. Functional enrichment analysis by clusterProfiler of PDR markers

Supplementary Figures

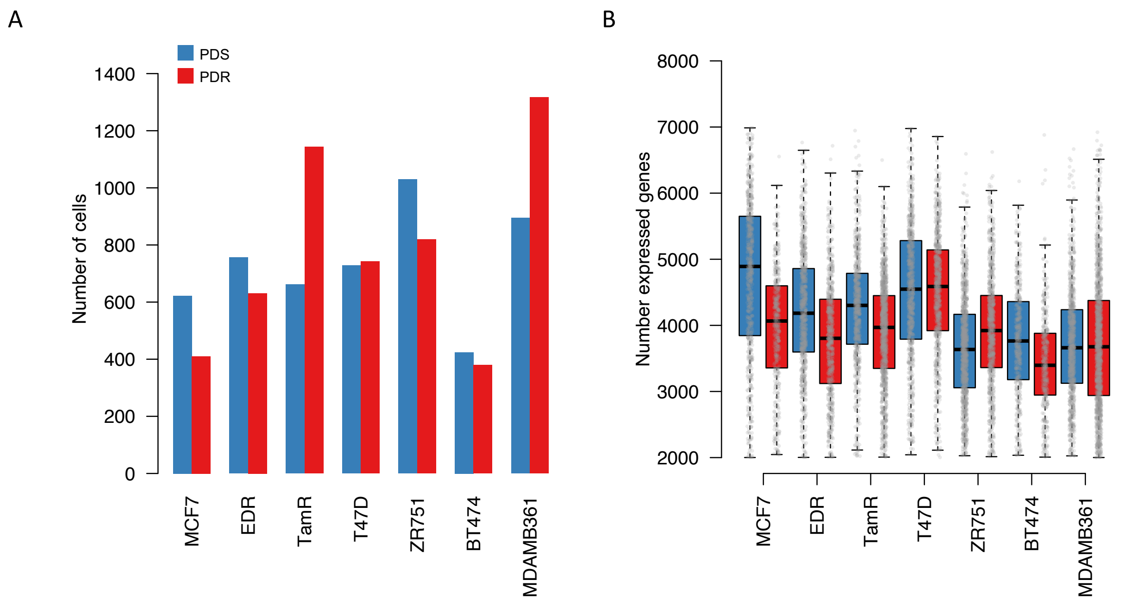

Supplementary Figure 1. A) Bar plots showing the distributions of the number of cells across the different cell lines. B) Box plots showing the distribution of the number of genes per cell across across the different cell lines.

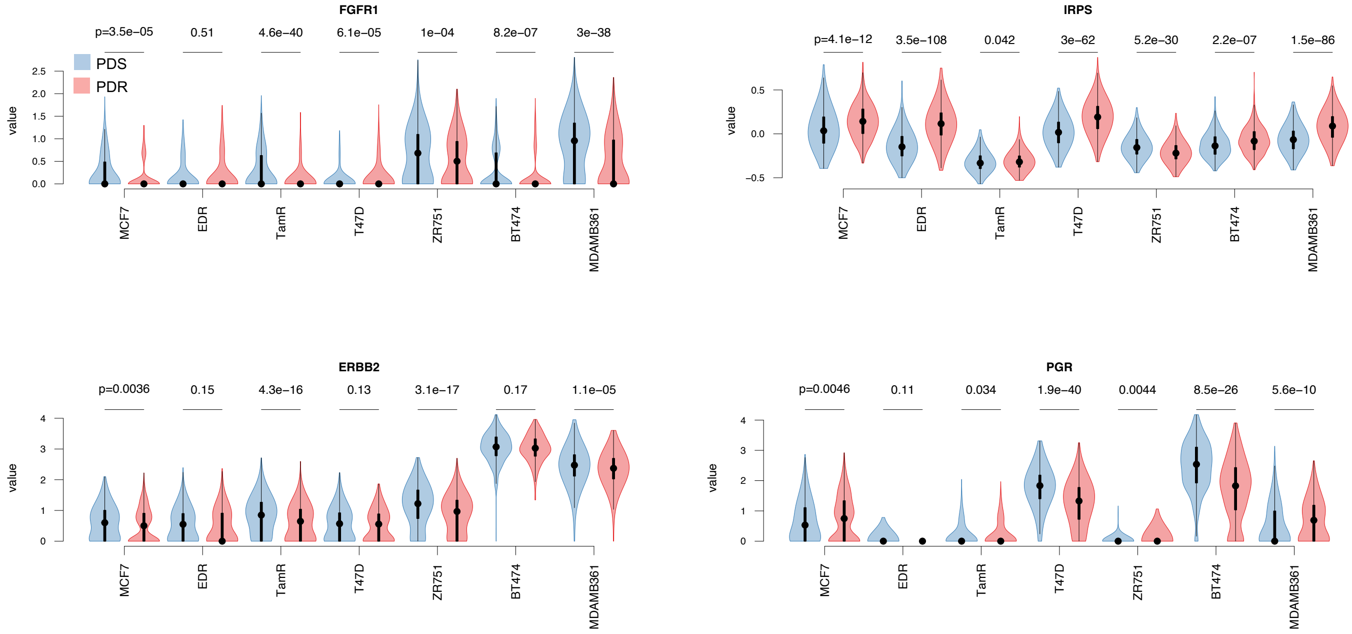

Supplementary Figure 2. Violin plots showing the distribution of FGFR1, IRPS signature, ERBB2 and PGR in sensitive (blu) and resistant (red) cells across the different cell lines. P values are estimated by Wilcoxon test

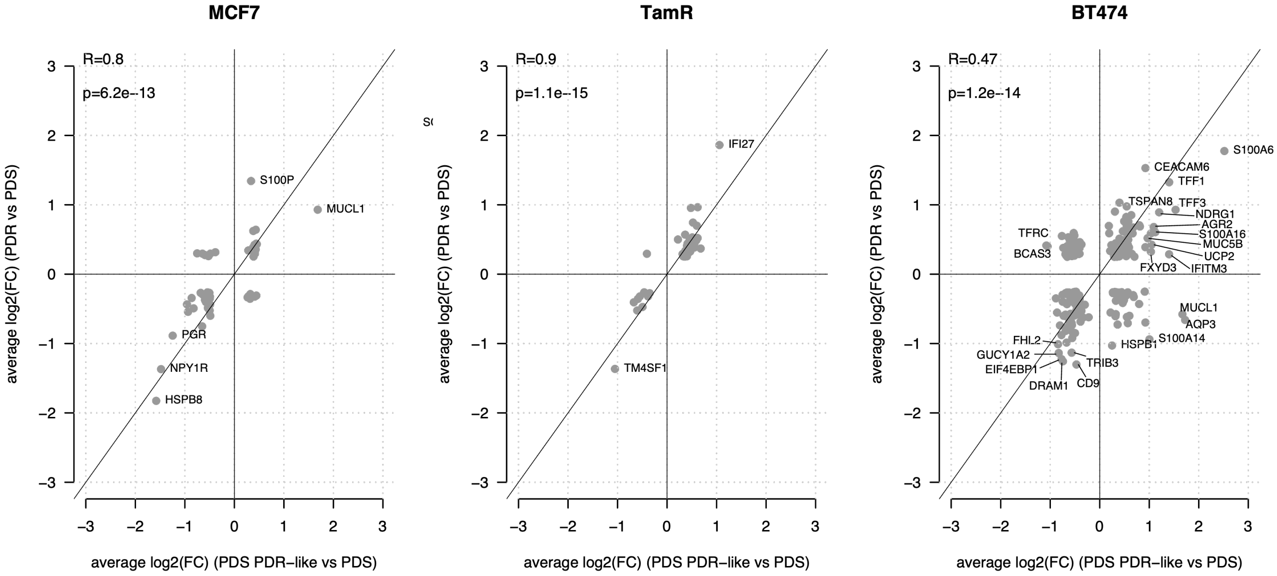

Supplementary Figure 3. Scatter plots showing average log2 Fold-change between differentially expressed genes in PDR vs PDS and PDS PDR-like (according to OLS) vs. PDS cells in the MCF7, TamR and BT474 models. Shown are genes having absolute log2 Fold-change > 1 in PDR vs. PDS or PDS and PDS PDR-like vs. PDS comparisons. R and p values are estimated by Pearson’s correlation test.

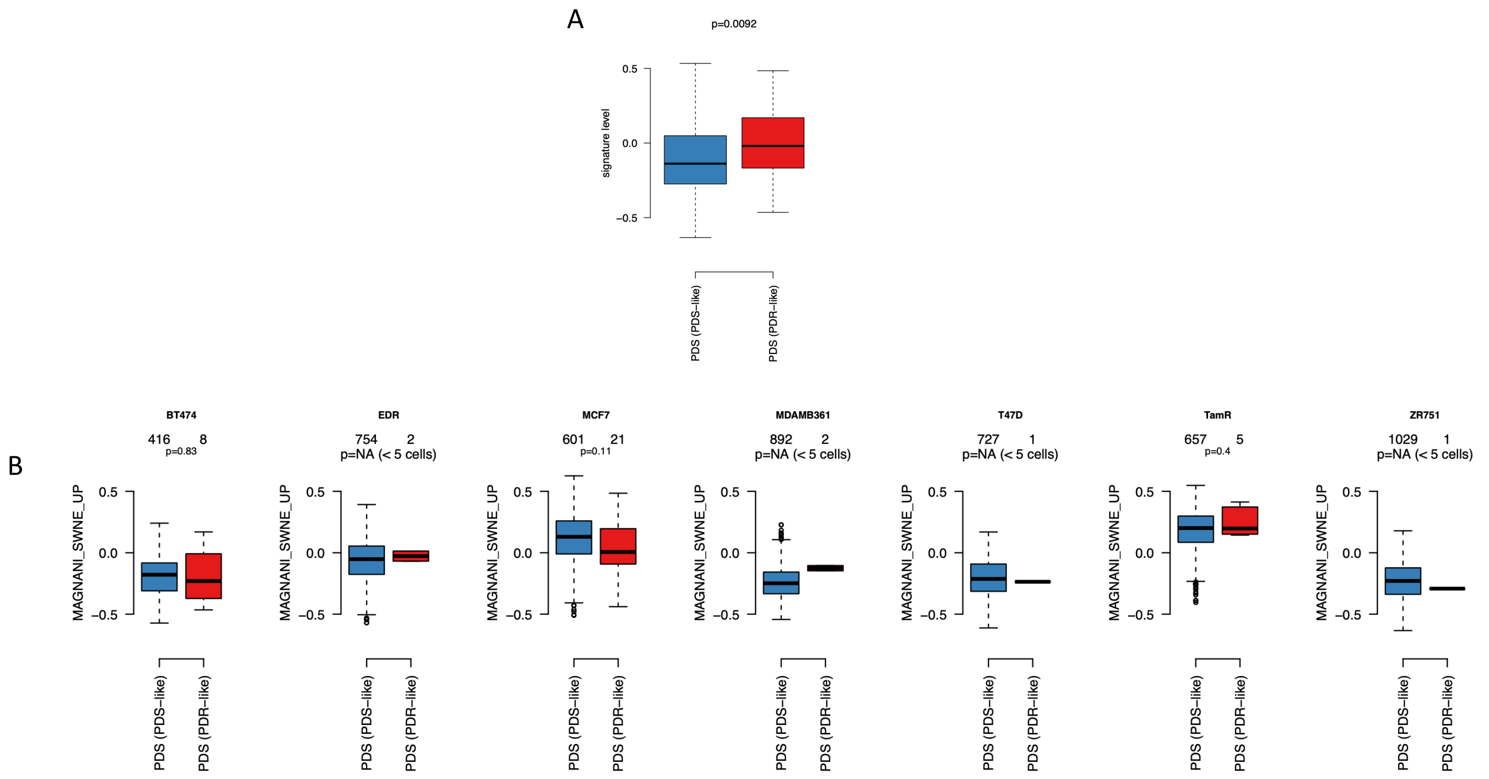

Supplementary Figure 4. Box plots showing the levels of the pre-adaptation signature, derived from the work of Hong SP and colleague (Magnani_SWNE_UP), in the PDR-like and PDS-like cells within parental models, both considering all models together (A) or separately (B). P-values are estimated by Wilcoxon test and only for cell lines having at least 5 PDR-like cells.

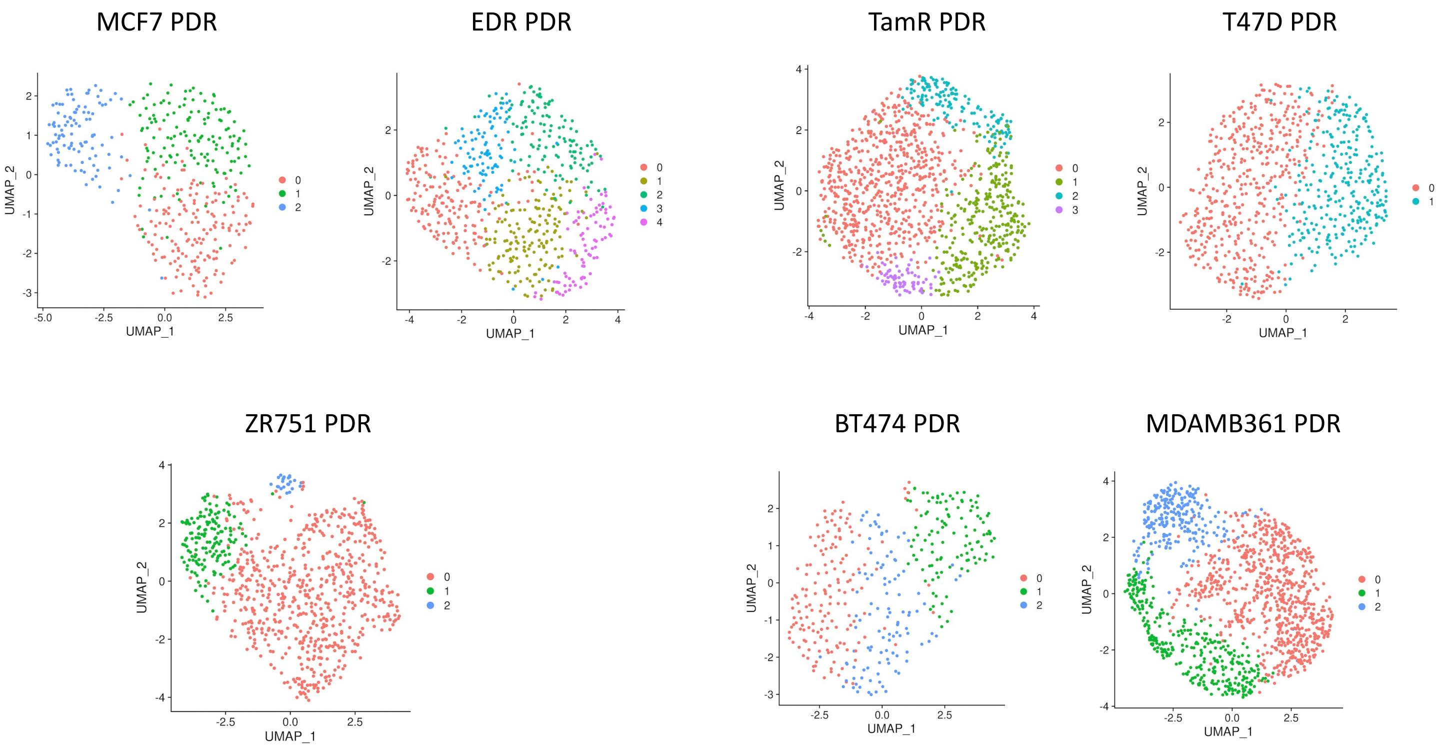

Supplementary Figure 5. UMAPs showing the transcriptional clusters (T-cl) of PDR cells in all models

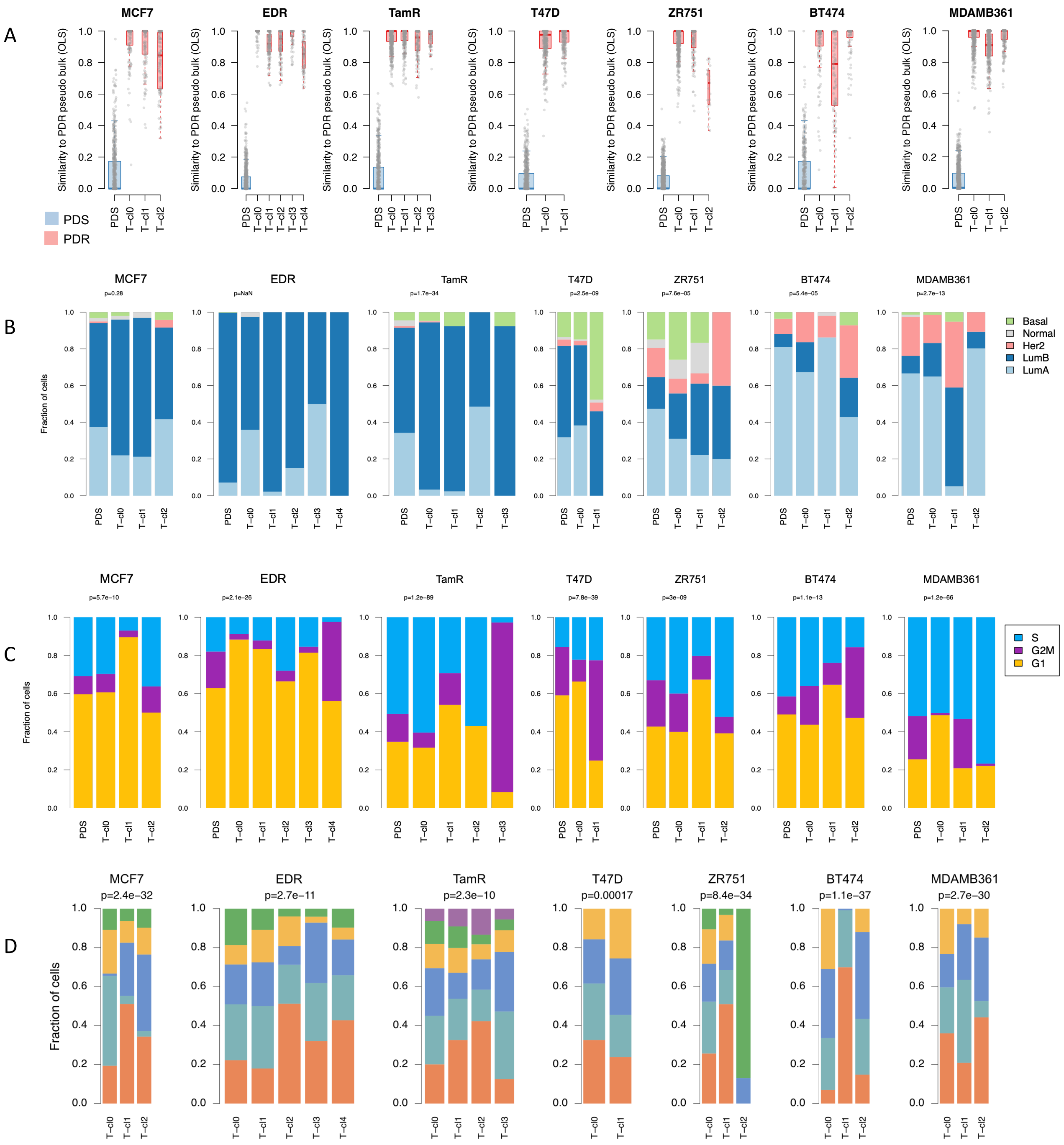

Supplementary Figure 6. A) Box plots showing the distribution of the similarity to the cell-type specific differentially expressed genes derived from the pseudo-bulk estimated by OLS in PDS (blue) and PDR (red) cells divided according to transcriptional clusters. B) Bar plots showing the distribution of PAM50 molecular subtypes in PDS cells and in

PDR cells divided according to transcriptional clusters. C) Bar plots showing the Distribution of the cell cycle phases in PDS cells and in PDR cells divided according to transcriptional clusters. D) Bar plots showing the distribution of copy number clusters in PDR cells divided according to the transcriptional clusters. P values are estimated using Chi square test.

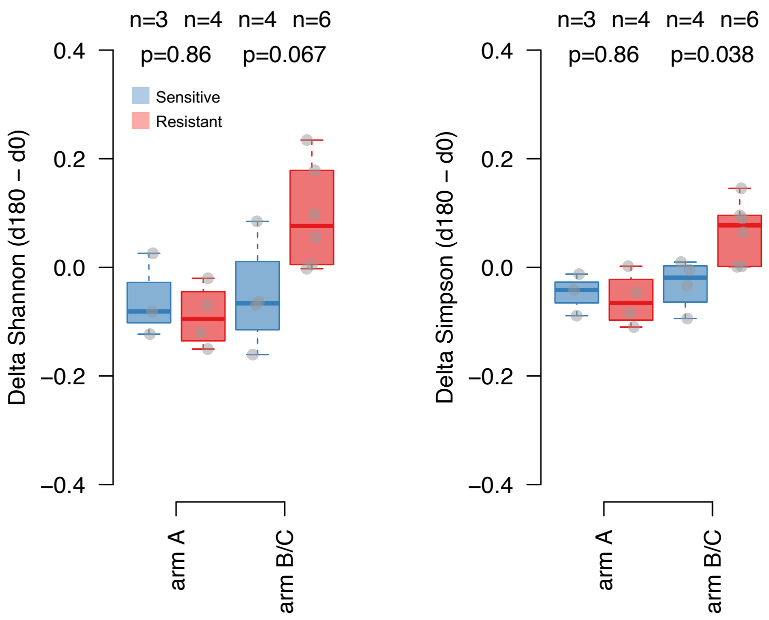

Supplementary Figure 7. Box plots of the delta Shannon and delta richness at day 180 compared to day 0 divided according to patient response to letrozole (arm A) or ribociclib and letrozole, independently of schedule (ARM B+C). Red= resistant patients, blue= sensitive patients. P values are estimated by Wilcoxon test**.**

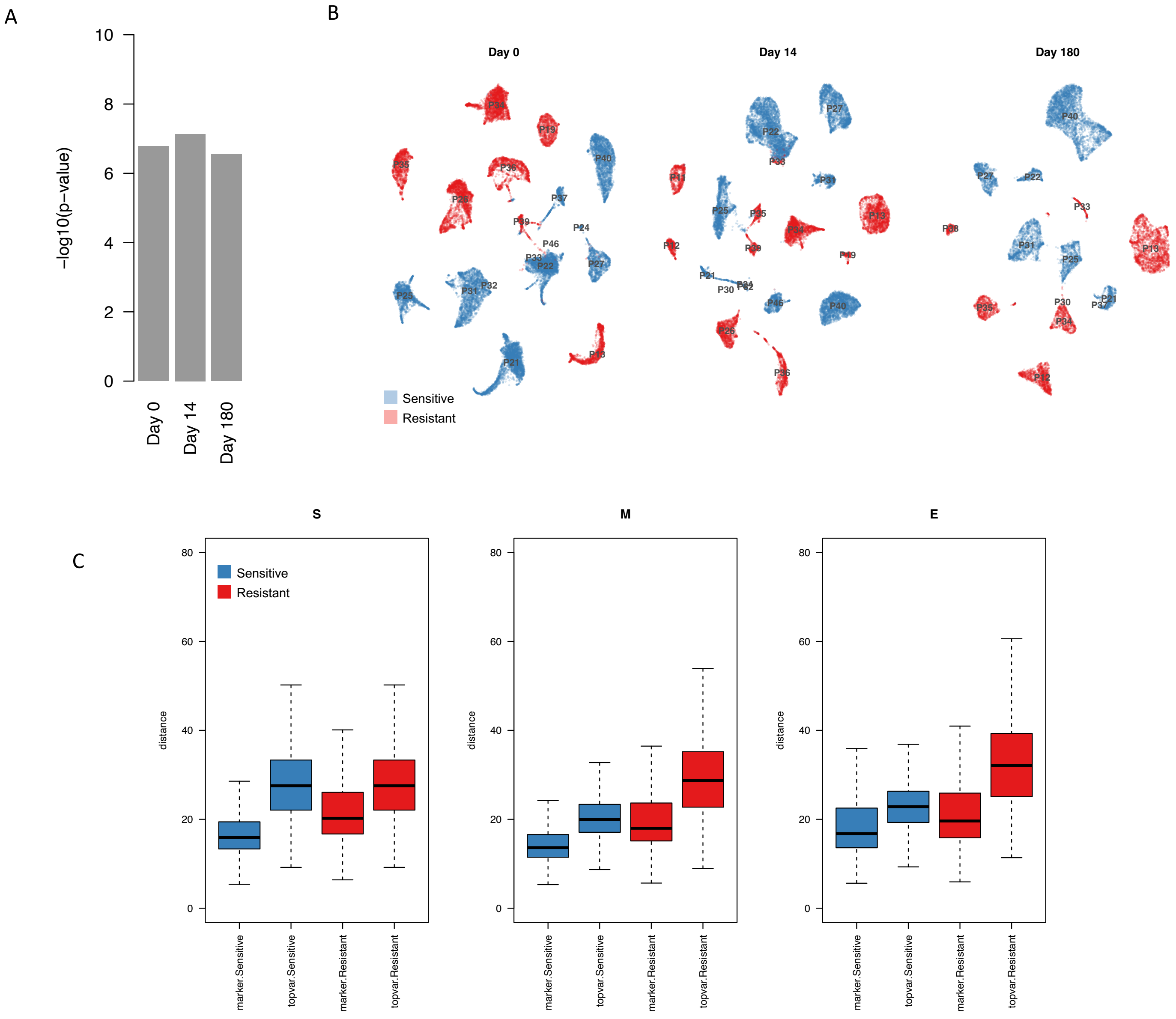

Supplementary Figure 8. A) bar plot showing the p-value of the enrichment by proportion test of the list of marker genes derived from our models in the list of differentially expressed genes in resistant versus sensitive samples derived from the FELINE dataset at each time point. B) UMAP visualization derived from the analysis of the top variable genes of cells from tumors of patients included in the FELINE trial and treated with ribociclib, independently of schedule (Arm B+C) at day 0, day 14 and day 180. C) Box plots showing the euclidean distance, using the PDR markers (marker) or the randomly selected genes of the same size as PDR markers (topvar) among cells deriving from

patients sensitive or resistant to ribocliclib and letrozole included in the FELINE trial, independently of schedule (Arm B+C) at day 0 (S), day 14 (M) and day 180 (E). Red= resistant patients; blue= sensitive patients.
